# Strategies for converting turn-motif and cyclic peptides to small molecules for targeting protein–protein interactions

**DOI:** 10.1039/d3cb00222e

**Published:** 2024-02-16

**Authors:** Deanne Hayward, Andrew M. Beekman

**Affiliations:** a School of Pharmacy, University of East Anglia, Norwich Research Park Norwich Norfolk NR47TJ UK A.Beekman@uea.ac.uk

## Abstract

The development of small molecules that interact with protein–protein interactions is an ongoing challenge. Peptides offer a starting point in the drug discovery process for targeting protein-interactions due to their larger, more flexible structure and the structurally diverse properties that allow for a greater interaction with the protein. The techniques for rapidly identifying potent cyclic peptides and turn-motif peptides are highly effective, but this potential has not yet transferred to approved drug candidates. By applying the properties of the peptide–protein interaction the development of small molecules for drug discovery has the potential to be more efficient. In this review, we discuss the methods that allow for the unique binding properties of peptides to proteins, and the methods deployed to transfer these qualities to potent small molecules.

## Abstract 2

Peptides are great starting points for drug discovery, but challenges remain in converting that potential to approved drugs. We review strategies for moving peptide features to small molecules, enabling small molecule drug development expertise.

## Graphical abstract

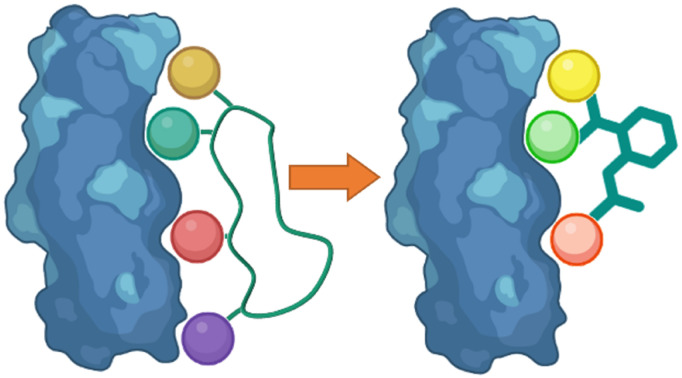

## Introduction

Proteins are the elementary unit to life with nearly all cellular functions being controlled *via* protein–protein interactions (PPIs).^1^ The release of human genome sequences catapulted PPI targeting to the forefront of chemical biology by providing a comprehensive map of our genes and subsequently protein interactions suitable for drug development.^2^ Although plentiful in suitable interaction targets, PPIs were largely deemed “undruggable” owing to the difficulty to control large interfaces that are flat, hydrophobic, and absent of binding pockets.^3^

Peptide-based therapeutics combat these challenges and target PPIs with remarkable potency, selectivity, and low toxicity.^4^ Peptides are larger than conventional small molecule binders and more structurally flexible, so cover a larger surface area of the PPI interface. The area of the protein that is involved in the interaction is often localised to small preorganized structurally defined regions recognized by a binding partner. Their structurally diverse peptide chains with various amino acids also encourages extensive hydrogen bonding and electrostatic interactions, contributing to the stability of the peptide–protein complex.

**Fig. 1:**
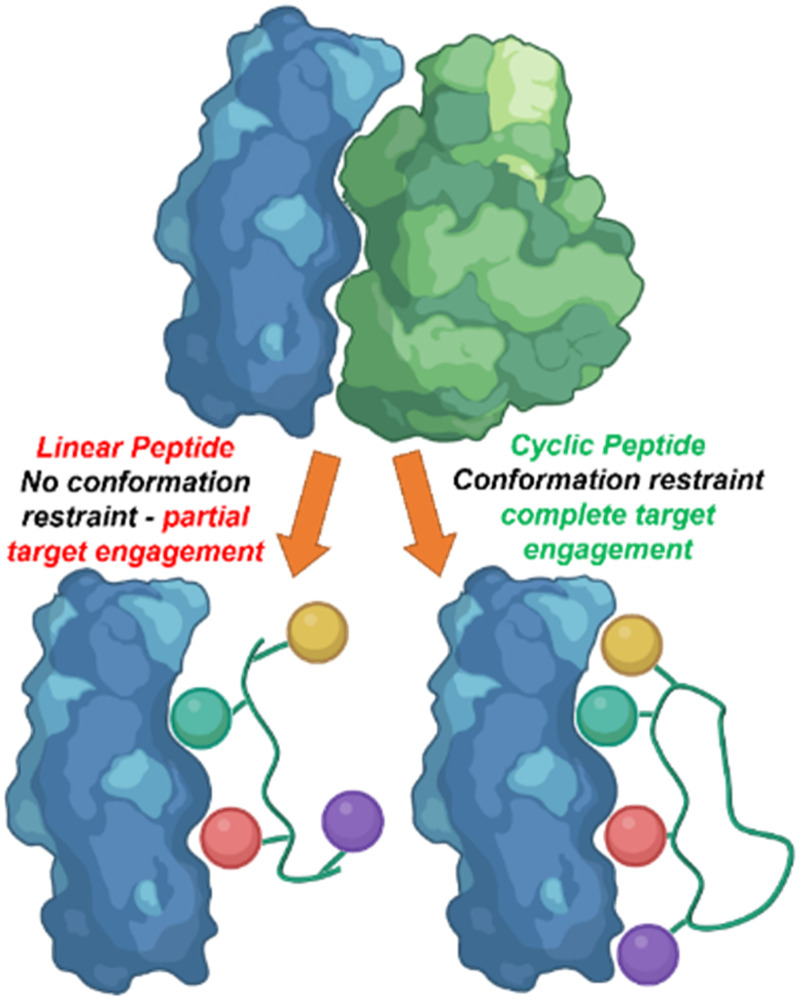
The rigid conformation of cyclic peptides increase peptide–protein interaction in comparison to linear peptides.

Linear peptides however, commonly demonstrate low oral bioavailability, poor metabolic stability, and short circulation time properties.^5^ Cyclic peptides, due to their more rigid conformation can overcome these limitations. The ring structure of the cyclic peptide allows for preorganization and the restricted conformation lowers the entropic cost upon binding resulting in increased binding affinity and specificity (Fig. 1).^6^

Cyclic peptides often lack amino and carboxyl termini, resulting in increased resistance to proteolysis. These features may also increase the ability to cross the cell membrane due to intramolecular hydrogen bonding within the ring.^7^ Cyclic peptides have the potential to mimic biologically relevant secondary structures of protein interactions (beta turns, alpha helices *etc.*) functioning as an inhibitor by mimicking and displaying these motifs.^6^ Pre-organization of these secondary structures can result in increased binding affinity.^8^

Cyclisation of peptides is categorized into four classes: side chain to side chain, head to tail, head to side chain, and side chain to tail. The point of cyclisation relates directly to the conformational constraint, influencing flexibility, secondary structure display and binding.^9–11^ Over the past two decades, 21 cyclic peptides have been successfully approved for clinical use cementing their importance within the pharmaceutical sector.^12,13^ Common approaches to identify cyclic peptides include natural product elucidation, protein sequence-based drug design, and display technologies. Isolating natural cyclic peptides as scaffolds for affinity development is promising due to their likelihood for biological activity. Difficulties in purification are common with natural isolation, often leading to analogues being optimised and prepared using solid phase peptide synthesis (SPPS).^14^ Peptides designed from amino acid sequences extracted from the interface of protein partners highlights “hotspot” sequences that often occur in the same secondary structure. To optimize potency and selectivity cyclisation techniques are utilized on these linear peptides.^15^

Several display techniques have been developed to discover cyclic peptides including phage display, split-intein circuit ligation of peptides and proteins (SICLOPPS), and random nonstandard peptides integrated discovery (RaPiD) system. Phage display is one of the most effective technologies to generate large libraries of peptide, proteins and antibodies based on the phage phenotype and genotype being physically connected. The cyclic peptide sequence is displayed on the phage coat protein and screened against target proteins to detect interactions. Recent developments have reported bicyclic peptides prepared by phage display.^16^ SICLOPPS utilizes ribosomal protein synthesis and splits an intein domain, the machinery which performs protein splicing, with cyclic peptide sequences. The first amino acid is required to be a nucleophilic cysteine or serine, while there is no limit on the number of amino acids in the target peptide. The intein domains come together, resulting in splicing of the cyclic peptide^17^ Similar to phage display in the genotype–phenotype linkage, the RaPiD system uses mRNA encoded libraries of peptides, with an additional custom-reconstituted translation system known as flexible *in vitro* translation (FIT) system enabling reprogramming of the genetic code with unnatural amino acids.^18,19^

Although peptides have opened an avenue into targeting and inhibiting PPI's, small molecules are still the leading compounds found on the drug market.^20^ The development of small molecules to target PPIs remains a critical task. Most small molecule design approaches tend to be focused on structure activity relationships (SAR), of large libraries of compounds based on computational modelling and refining the structures until optimum affinity is achieved. The functionality of using peptides as valuable starting points for drug development could make this process more efficient due to their modular structure, variable functional groups and ease of preparation.^21^

Despite the rapid rise in effective cyclic peptide identification techniques for controlling PPIs, methods to transfer these characteristics to small molecules are limited. Few studies have optimised the structural data found about the superior binding of cyclic peptides (or even turn motifs) to PPIs, analysed the essential amino acid side chain properties that create binding and applied that knowledge to a small molecule structure, avoiding the laborious journey of SAR or expense and time of testing large libraries. However, when applied there have been several successful technologies capable of transferring the characteristics of peptide binders to non-peptidic small molecules.^22^

When developing a peptidic inhibitor for a PPI, structural biology is pivotal. Commonly, these projects are supported by X-ray crystallography, protein-based nuclear magnetic resonance (NMR) spectroscopy and cryogenic electron microscopy.^23^ Further information can be obtained through ongoing monitoring with lead compounds revealing any conformational changes that may occur upon binding. It is important to note that not all interactions drive the affinity of the PPI, and determining which interactions are essential for binding or inhibition is invaluable information.

When structural information is absent interactions can be investigated experimentally using an alanine scan, a process which takes a peptide sequence of interest and sequentially mutates each amino acid residue with alanine^24^ Depending on whether inhibition is lost or remains, determines which amino acid residue or residues are key for this interaction to take place leading to targetable “hotspots”. Once key fragments for binding are identified, the conformation of the peptide can be investigated by introducing constraints at various positions to decrease conformational flexibility.

In this review, small molecules that have been designed and modified using structure activity relationship information from cyclic or turn based peptides will be discussed. Three strategies have emerged for the transfer of binding interactions of peptides to small molecules. (1) The decoration of small molecule scaffolds with amino acid side chain functionality. This method sees the direct addition of amino acid side chains to non-peptidic skeletons, and subsequent refinement for ideal functional group placement. (2) The mimicking of peptide side chain interactions with similar functionality. This method applies the addition of functional groups which can emulate amino acid side chain interactions to more synthetically accessible small molecules. (3) The enhancement of current small molecule protein binders using structure activity relationship information from peptide binders. This approach modifies known small molecule binders with the amino acid side chain functionality.

### Transferring peptide side chain functionality to small molecule scaffolds

**Fig. 2:**
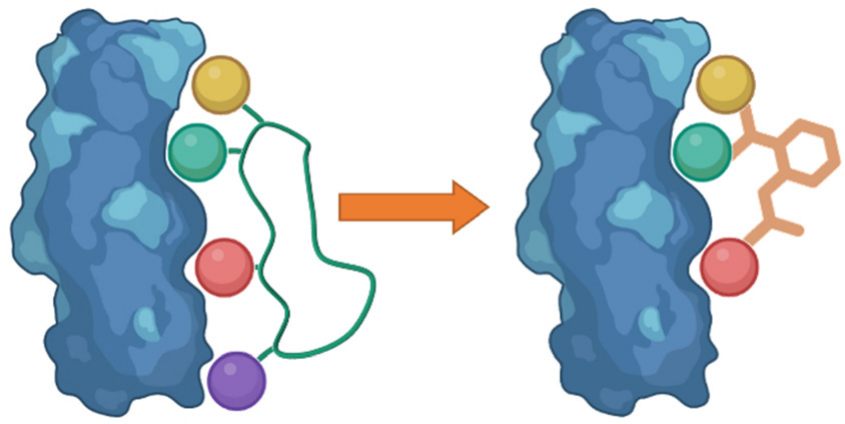
Taking the side chain functionalities of the peptide and applying them to small molecule skeletons.

Following the identification of peptide sequences capable of controlling the targeted interaction, the essential amino acid side chains can be extracted. These side chain functionalities can form part of very short peptides or can be placed on non-peptide skeletons (Fig. 2). Small molecule scaffolds should be small and rigid, aiding in preorganization of side chains that are important for activity.

**Fig. 3:**
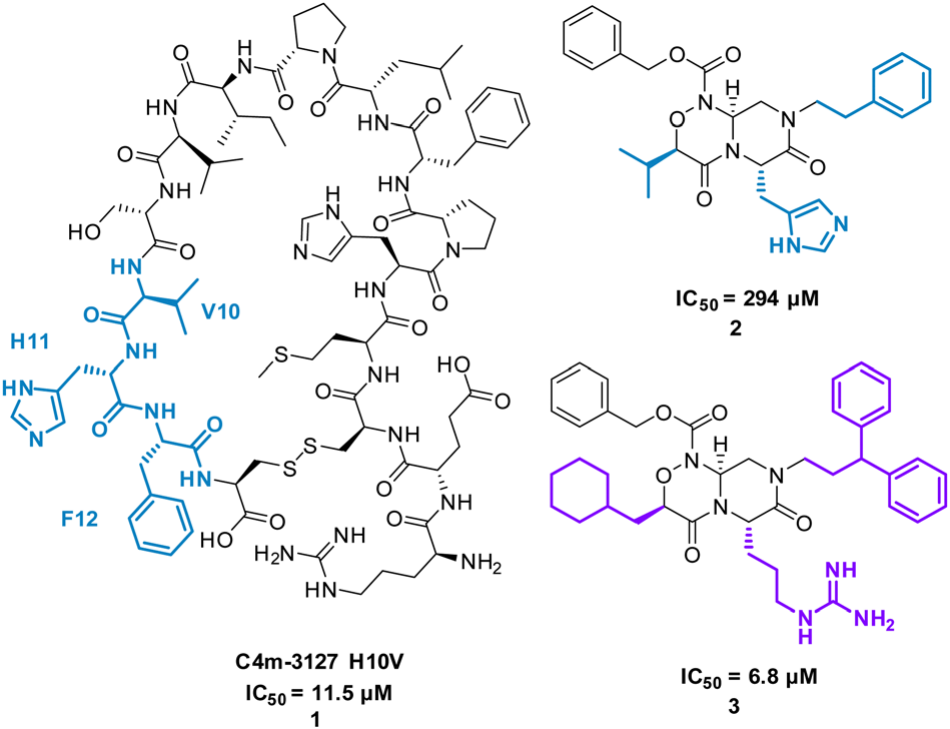
Structures of cyclic peptide 1 and small molecules 2 and 3.

The cyclic peptide C4m-3127 H10V (MHPFLPIVSVHF) 1 (Fig. 3) inhibits the interaction between Cytotoxic T-lymphocyte-associated protein 4 (CTLA-4) and B7-1 (IC_50_ of 11.5 μM) and was identified using protein synthesis using recombinant elements (PURE).^25^ CTLA-4 is expressed on the surface of activated T cells and binds with ligands B7-1 and B7-2, suppressing T cell activation.^25^ Alanine scan mutagenesis highlighted eight mutants (H2, P3, L5, P6, I7, S9, H11, and F12) led to a decrease in binding with CTLA-4. In the absence of structural data, the team computationally modelled consecutive trimers of amino acid side chains onto 40 virtual scaffolds, highlighted previously by Takashima *et al.* in their development of “Pepmetics”,^26^ and performed molecular docking of these compounds to CTLA-4. Tsuihiji *et al.* proposed that three consecutive residues imprinted on a small molecule skeleton would emulate a β-turn, a common binding motif in cyclic peptides, and hypothesised to be responsible for the binding of 1. Several compounds were highlighted for synthesis, leading to compound PGF00432 2 (IC_50_ of 294 μM) which mimicked residues V10, H11 and F12 (Fig. 3). Structure based drug design, complemented by the docking pose of 2 suggested the replacement of histidine with arginine, the replacement of valine with a cyclohexylmethyl group, and the replacement of phenylalanine by 3,3-diphenylpropyl resulting in compound PGF00506 3 with an IC_50_ of 6.8 μM (Fig. 5).^20^ Competitive ELISA was performed on 3 and peptide 1 to confirm 3 binds to CTLA-4 on the same binding surface as 1. The arrangement of the benzyl, cyclohexyl and arginyl side chains in 3 are predicted to mimic the β-turn arrangement of V10, H11 and F12 in the original peptide. 1 is a successful small molecule that is a direct mimic of cyclic peptide 3 with an increased inhibitory activity.

**Fig. 4:**
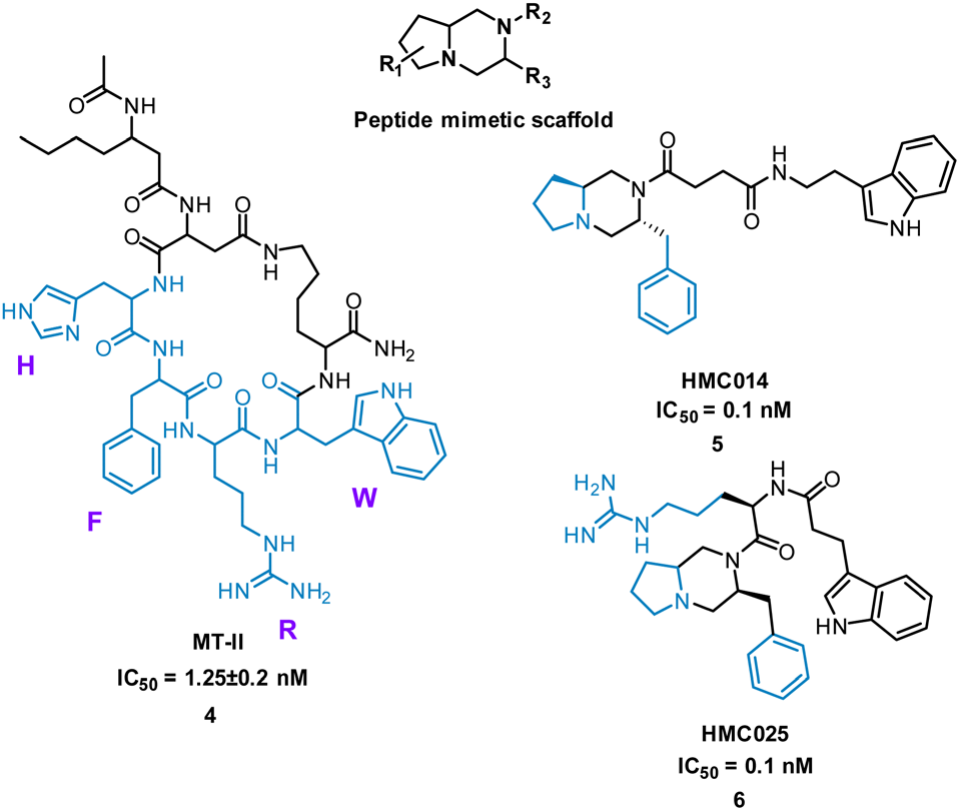
Structure of cyclic peptide 4, and small molecules 5 and 6.

Extensive research has taken place on peptides that modulate human melanocortin receptors (hMCRs) for various potential therapeutic values. Peptidic agonists of hMCRs all possess the sequence HFRW.^27,28^ Hruby *et al.* virtually screened a library of bespoke pyrrolopiperazines, decorated with functionality based on known peptide and small molecule ligands. Comparable to the HFRW sequence, previous small molecules required two hydrophobic aromatic groups and a basic nitrogen for activity.^29^ By overlapping the proposed compounds and MT-II, 4 (Fig. 4) the structures that best aligned with 4 were selected for synthesis and biological evaluation.^30^ Biological competition assays using hMCRs expressed in cells confirmed displacement of ^125^I-labeled NDP-α-MSH comparable to treatment with 4. Each small molecule showed binding to one or more of the receptors (melanocortin receptors 1–5, MC1-MC5) with most having high selectivity. Notably, five new antagonists with sub-molar IC_50_ values were identified for the relatively unexplored MC-5 receptor, including 5 and 6 (Fig. 4).^29^

**Fig. 5:**
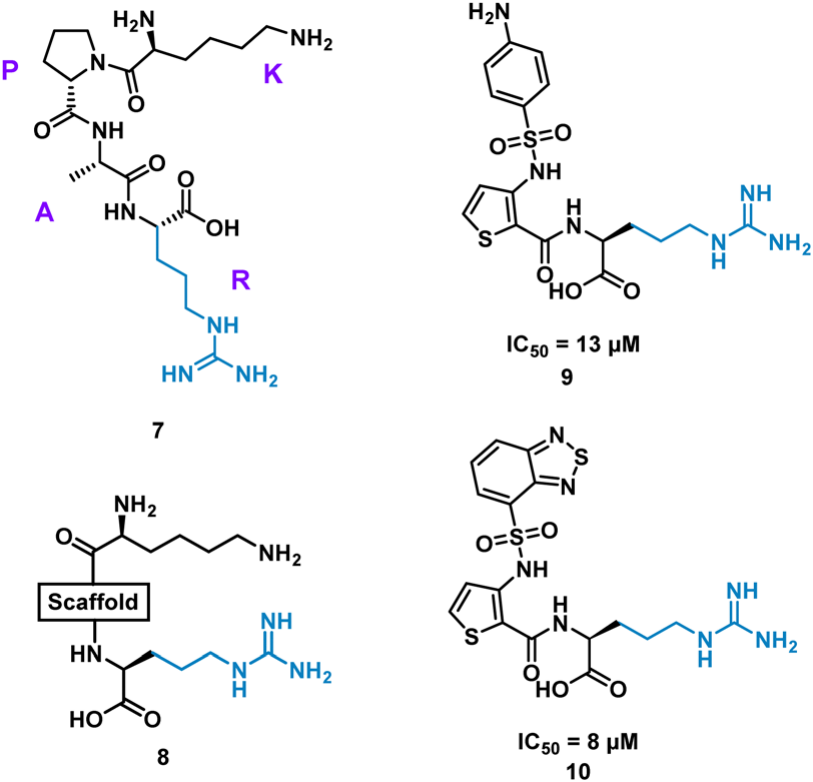
Structure of turn-motif peptide 7, and small molecules 8, 9, and 10.

Inhibition of the interaction between receptor neuropilin (NRP1) and vascular endothelial growth factor (VEGF-A) impacts tumour growth, and has been shown to synergistically enhance VEGF-A inhibitors.^31^ Using the crystal structures of NRP1/VEGF-A and an alanine scan of the identified binding site, the VEGF-A amino acids Tyr297, Trp301, Thr316, D320, S346, T349, Y353, K351 and Trp411 were highlighted as essential for binding. The turn motif peptide KPAR 7 (Fig. 5) was shown to bind VEGF-A and inhibit NRP1 interaction, with Arg and Lys essential for activity.^32,33^ Using modelling, a series of scaffolds to connect the terminal arginine to lysine, or a lysine mimic, were screened (8). The best scaffold identified was thiophene-based, exemplified by 9, with an aminophenylsulfonamide lysine mimic, demonstrating an IC_50_ of 13 μM. Lead development culminated in a benzothiadiazole heteroaryl improved 10 (IC_50_ = 8 ± 0.02 μM) (Fig. 5). X-ray crystallography showed, importantly, the arginine residue fits almost perfectly into the binding pocket, and a critical intramolecular bond between the carbonyl amide NH and the sulfonamide nitrogen aided in compound stability. 10 showed inhibition of VEGF-A/NRP1 but not to VEGFR2 expressing cells and showed inhibition of VEGF-A binding to lung carcinoma and prostate carcinoma cells both which express NRP1. 10 is a successful mimicked structure of a turn motif peptide 7, with the ability to inhibit VEGF-A binding in carcinoma cells, providing a small molecule for potential anti-NRP1 drug.^34^

**Fig. 6:**
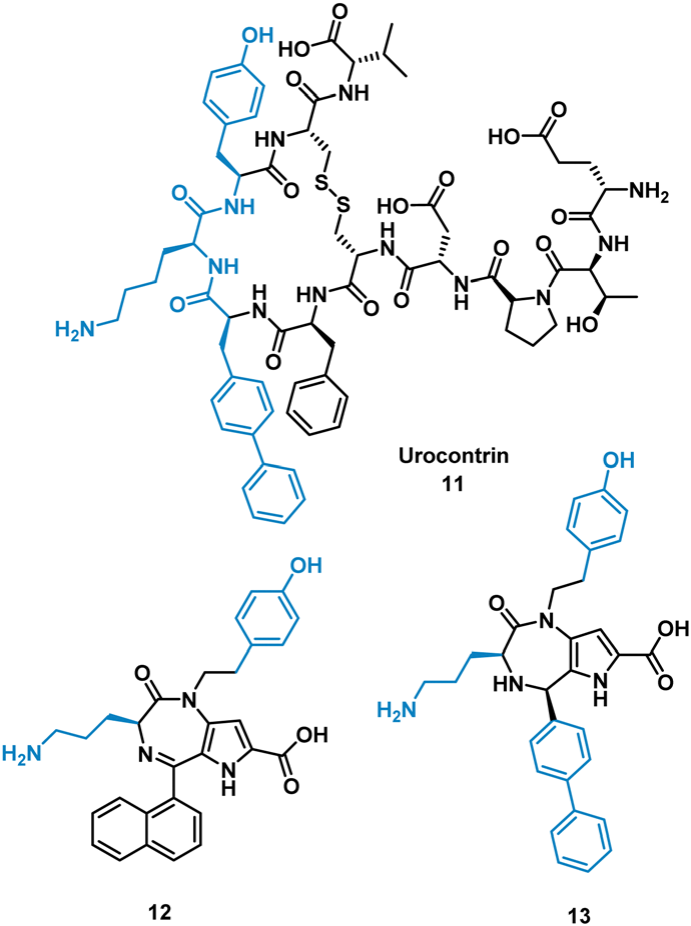
Structures of cyclic peptide 11, and small molecules 12 and 13.

The urotensin II receptor (UT) binds two endogenous cyclic peptide ligands, urotensin II (UII, ETPD-*c*[CFWKYC]-V) and urotensin II-related peptide (URP, A-*c*[CFWKYC]-V). The urotensinergic system regulates various organs and tissue and has shown to be a promising target for cardiovascular diseases. The WKY residues are important for binding in both UII and URP, but URP lacks the N-terminal and therefore likely binds differently.^35–37^ Currently the market lacks any agonist or antagonist for this interaction with the ability to discriminate between UII or URP activity. Previous SAR modelling suggested that Trp interacts with the UT receptor *via* polar interactions rather than hydrophobic interaction. By replacing Trp for a biphenylalanine (Bip), resulting in urocontrin, 11 (Fig. 6), the peptide is more potent and selective, likely through advantageous hydrophobic interactions, inhibiting the response of UII (aortic ring contraction of UII = 116%, in the presence of 11 = 0%) with no effect on URP induced vasoconstriction (aortic ring contraction of URP = 132%, in the presence of 11 = 107%).^37^ Dufour-Gallant and co-workers sought to combine this selective cyclic peptide characteristics onto a small molecule scaffold, pyrrolodiazepinones.^38,39^ X-ray crystallography confirmed pyrrolodiazepinones have the ability to adopt the inverse γ-turn found in the binding motif of 11.^40^ A library of small molecule UT ligands were designed based on this concept, and mimicking the key Bip-Lys-Tyr binding motif, and tested *in vitro* using a competitive binding assay resulting in 12 and 13 (Fig. 6). 13 can selectively inhibit the vasoconstrictive effects of UII (aortic ring contraction of UII = 116%, in the presence of 13 = 0%), while having little effect on URP-induced vasoconstriction. 12 shows the opposite effect, selectively inhibiting vasoconstrictive effects of URP, while having little effect on UII induced vasoconstriction (aortic ring contraction of URP = 132%, in the presence of 12 = 46%; UII in the presence of 12 = 74%). Despite the low potency, the development of these non-peptide inhibitors mimicked from the original cyclic peptide have shown that 12 and 13 have different mechanisms of action with specific targeting to either UII or URP.^41^

**Fig. 7:**
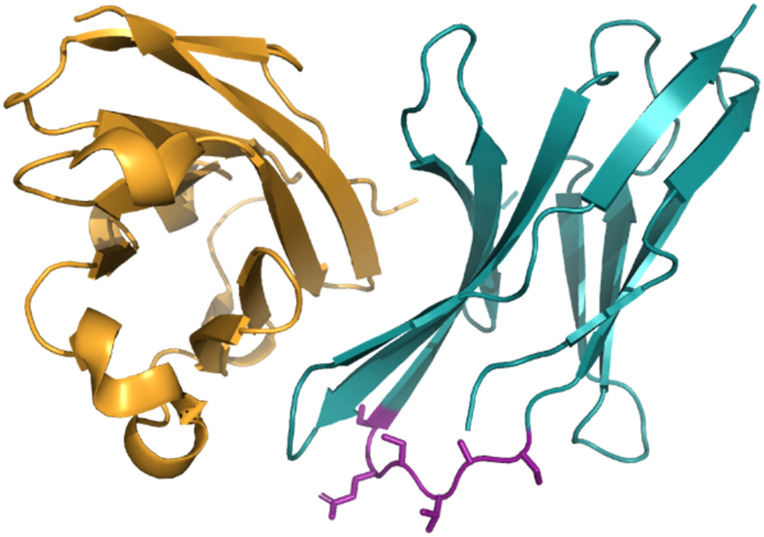
The PD-1/PD-L1 interaction highlighting the SNTSESF sequence in purple.

**Fig. 8:**
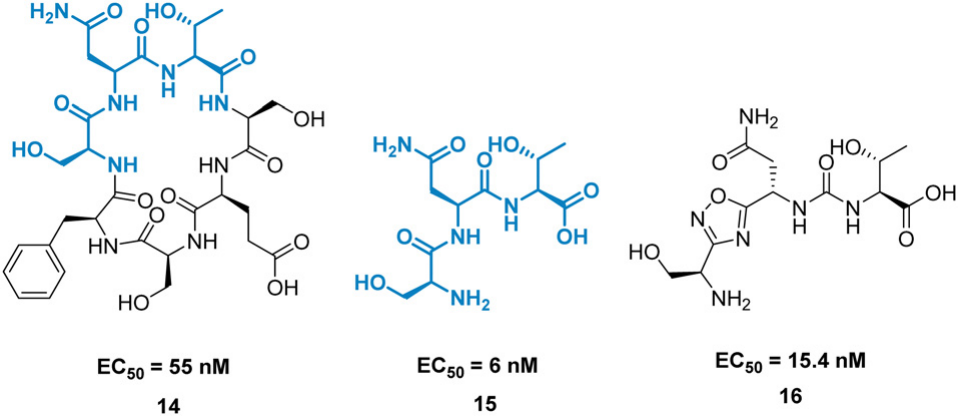
Structures of cyclic peptide 14, tri-peptide 15, and small molecule 16.

A high-profile example of this method is the development of CA-170, derived from peptide AUNP-12 (H-SNTSESFK(SNTSESF)FRVTQLAPKAQIKE-NH_2_).^42^ AUNP-12 was identified as a PD-1/PD-L1 inhibitor through the synthesis of a series of loops and strands from PD-1 at the PD-L1 interface. AUNP-12 was found to rescue T cells from inhibitory activity of PD-L1 in a mouse splenocyte assay to 100% at 100 nM. Truncation experiments highlighted the SNTSESF sequence as the source of activity (EC_50_ = 81 nM), despite not being present at the PD-L1 interface (Fig. 7, purple). This peptide demonstrated poor stability in plasma, but head-to-tail cyclisation resulted in 14 (Fig. 8), demonstrating a slight increase in activity (EC_50_ = 55 nM) and stability in mouse, rat, and human plasma.

The tri-peptide 15 SNT retained activity (EC_50_ = 6 nM), but again was highly unstable in plasma. CA-170, 16 was the culmination of several strategies to stabilise the peptidic backbone, incorporating a urea moiety, and a fused 1,2,4-oxadizole heterocyclic backbone. 16 maintained activity (EC_50_ = 15.4 ± 1.3 nM) while restoring stability in plasma, and has gone on to face clinical trials as an inhibitor of the immune checkpoint proteins PD-1/PD-L1.^43^

**Fig. 9:**
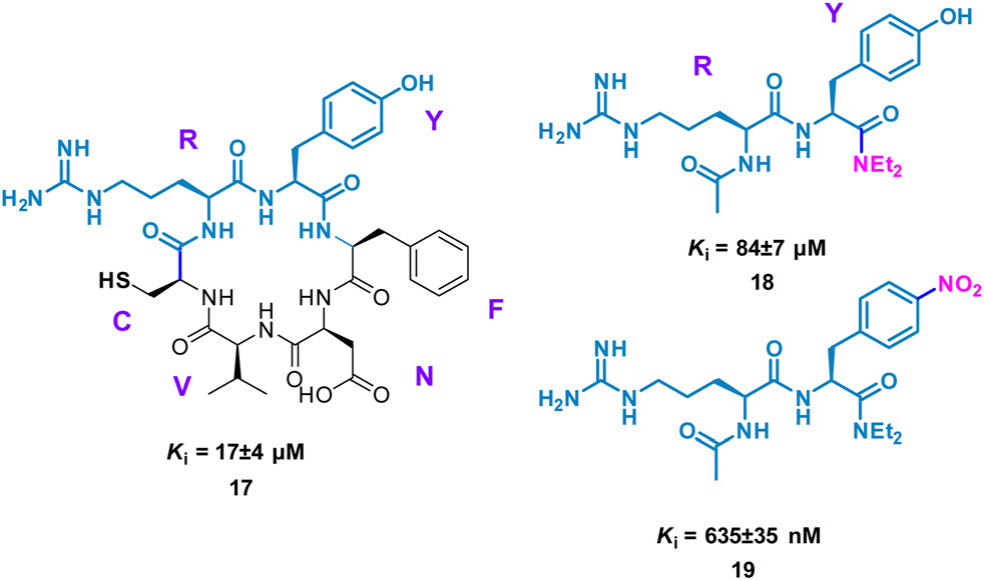
Structures of cyclic peptide 17, di-peptide 18 and small molecule 19.

Perhaps conceptually the simplest method for mimicking cyclic peptide residues for binding is to create di- or tri-peptides of the key amino acids identified. From here, further SAR may be applied, modifying the side chains of the amino acids to enhance binding affinity. The Tavassoli group demonstrated this approach using a cyclic peptide (CRYFNV, 17, Fig. 9) inhibitor of ATIC (aminoimidazole carboxamide ribonucleotide transformylase/inosine monophosphate cyclohydrolase) homodimerization, a key interaction in *de novo* purine biosynthesis. This cyclic peptide, identified using a combination of SICLOPPS technology with bacterial reverse two-hybrid system (RTHS), was found to be a potent inhibitor (*K*_i_ = 17 ± 4 μM) of homodimerization.^44,45^ An alanine scan of the cyclic peptide identified the arginine and tyrosine residues as significant for inhibition. This resulted in dipeptide 18 (*K*_i_ = 84 ± 7 μM), which mimics the active RY motif of the cyclo-CRYFNV. Further structural modifications culminated in the addition of the electron withdrawing nitro group, resulting in a 120-fold improvement in activity with a *K*_i_ value of 685 ± 35 nM (Fig. 9). 19 maintained inhibition of ATIC homodimerization, in the presence of bovine serum albumin (BSA), suggesting specificity as a cyclic peptide mimic.^46^

**Fig. 10:**
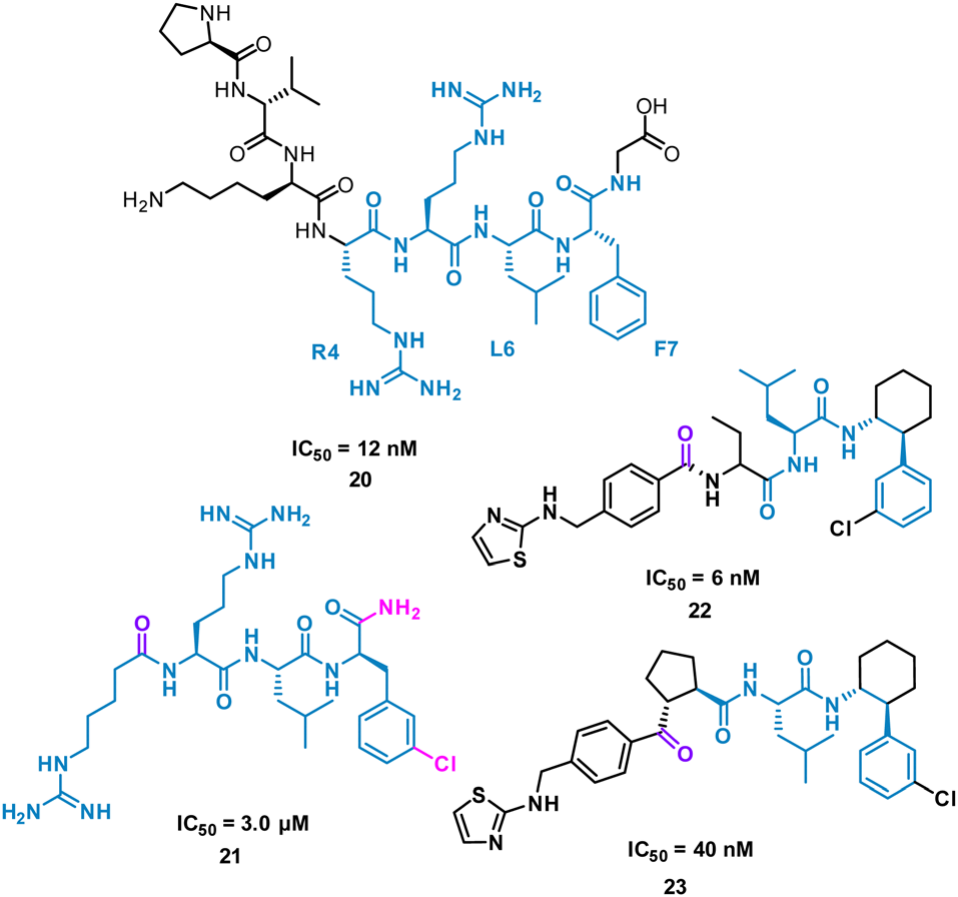
Structures of turn-motif peptides 20 and 21, and small molecules 22 and 23.

The turn-motif peptide PVKRRLFG 20 (Fig. 10) is a Cyclin A binder that was derived from the crystal structures of the protein–protein interactions of Cyclin A and a variety of protein partners, including E2F1, p27, p53, and p101.^47,48^ An alanine scan highlighted the essential residues, R4, L6 and F7 which informed truncated peptide design. Investigation of the crystal structure of a related peptide (KPSAC**R**N**LFG**P, PDB: 1H27) highlighted structural modifications for further investigation.^49^ To mimic the preorganization of peptide 20, which holds R4, L6 F7 on the same face and provide the peptide binding surface (represented in 2D in Fig. 10), modification began by focusing on the turn conformation of the RNLF motif upon binding with modifications leading to 21 as a starting tetra-peptide (Fig. 10). Structure activity relationship (SAR) studies highlighted 22 which was 500-fold more potent, contains fewer rotational bonds and is neutral at physiological pH. However, the peptidic nature of 22 was susceptible to metabolism and offered poor oral absorption, and so amide bonds were removed.

The N-terminal amide of 22 was replaced by a ketone to exploit a key H-bond with the protein, along with incorporation of a cyclopentylbackbone, resulting in in 23 with an IC_50_ of 40 nM.^50^

23 showed moderate inhibition of cyclin A and when modelled onto a crystal structure of the peptide fragment RNFL showed the ketone to accept the hydrogen bond similarly to the N-terminal amide carbonyl.

### Using peptide side-chain functionality to inspire small molecule design

**Fig. 11:**
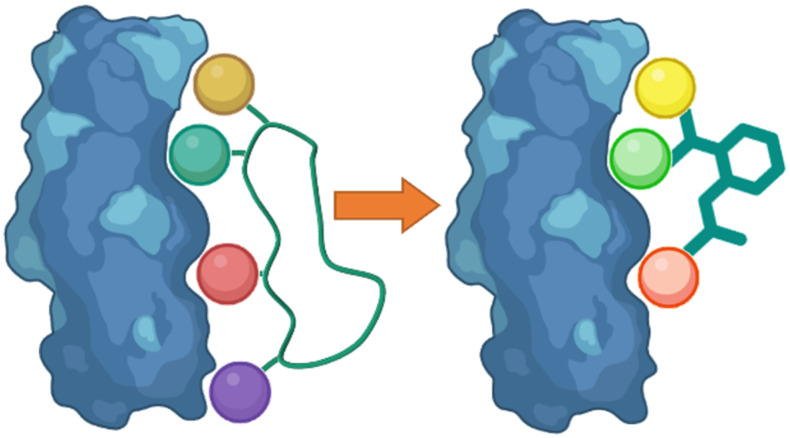
Designing small molecules that mimic side-chain properties of peptides.

The analysis of a peptide can also be used to influence design of small molecules through important properties rather than direct structure mimicking. This is used when there is bountiful knowledge about peptides that bind to the PPI but maybe no known small molecule that has shown promise. Characteristics including sterics, hydrogen bonding and lipophilic nature are analysed and applied when designing the small molecule (Fig. 11).

**Fig. 12:**
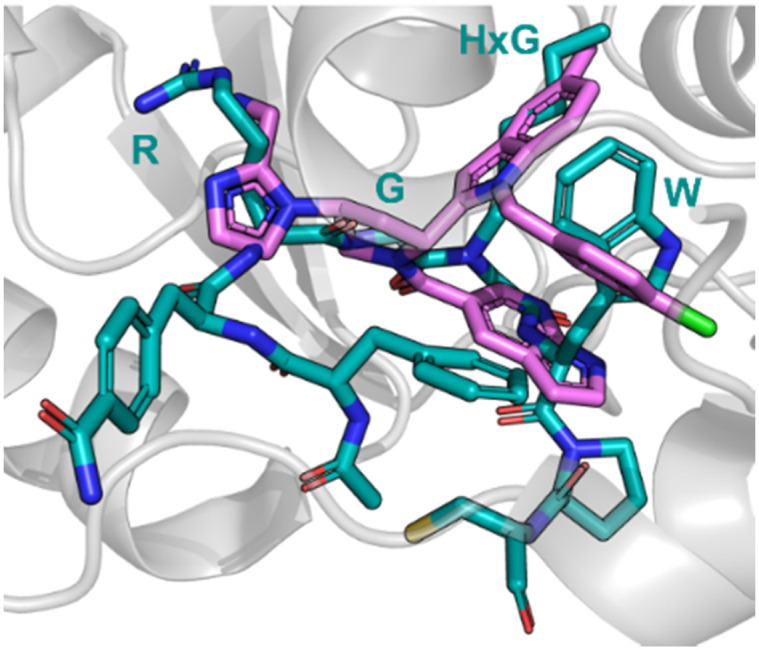
Peptide 24 (cyan, PDB: 7WMC) and 27 (magenta, PDB: 7WMT) bound to NNMT. Key binding residues R, G, HxG, and W are mimicked in 27 by pyrazole, indole, azaindole, and chlorophenyl substituents.

Nicotinamide *N*-methyltransferase (NNMT) is an intracellular enzyme over expressed in many diseases such as obesity, diabetes, and alcohol related liver diseases.^51–55^ Identified using mRNA display, 24 (macrocyclic peptide) exhibited good inhibitory activity for NNMT with an IC_50_ = 0.1 μM but lacked cellular potency. An alanine scan of peptide 24 showed side chains of R, G, HxG (*N*-hexyl-glycine) and W were essential and located at two crucial binding pockets (Fig. 12). Arg picks up electrostatic interactions with NNMT while HxG and Trp sat in the hydrophobic pocket of NNMT suggesting two lipophilic side chains are important for strong binding. HxG alongside Gly were also indicated to act as hydrogen bond acceptors to displace entropically unfavoured water molecules and restore electrostatic interactions with NNMT. These four key pharmacophore characteristics matching the four functional groups were carried forward into virtual screening to identify small molecules that possessed all key traits.

**Fig. 13:**
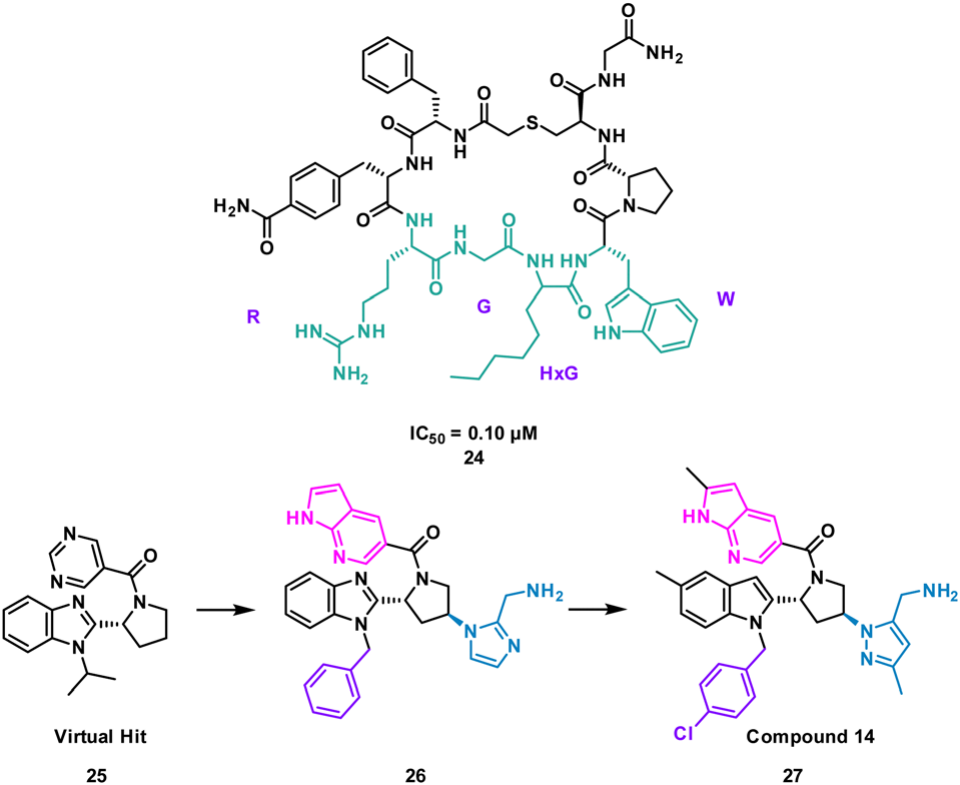
Structures of cyclic peptide 24 highlighting RGHxGW sequence (cyan), inspired hit 25, and subsequent small molecules 26 and 27.

Unfortunately, there was no compound hits that incorporated all four binding elements, therefore three features were chosen, two hydrogen bond acceptors and the hydrophobic properties to give virtual hit 25 (Fig. 12). To mimic the Arg electrostatic interactions, an imidazole with an amine functional group was introduced (Fig. 12, blue). A phenyl group was added to the benzimidazole for interactions with the hydrophobic pocket (Fig. 12, green). Addition of an azaindole group to form a polar interaction (Fig. 12, pink) resulted in 26 (Fig. 13). 26 showed weak inhibitory activity and NMR experiments confirmed binding to NNMT. SAR development yielded 27 showing a potent inhibitory activity (IC_50_ = 0.0011 μM) and promising cell based activity without toxicity (IC_50_ = 0.40 μM).^56^ Through peptide mimicking, a novel cell potent and selective small molecule was identified to inhibit NNMT without conventional library screening.

**Fig. 14:**
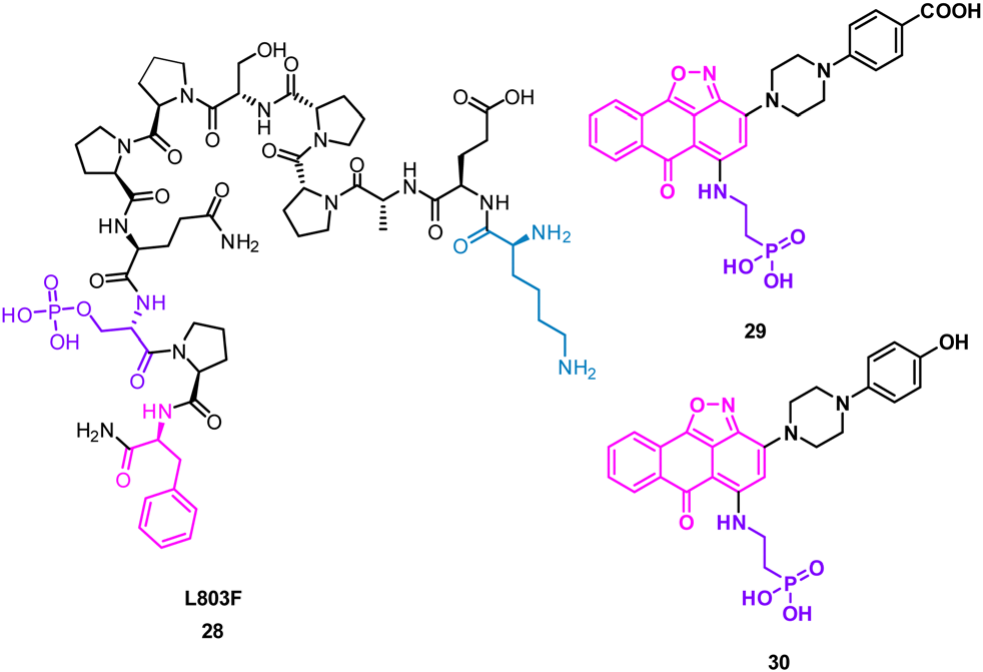
Structures of turn-motif peptide 28 and small molecules 29 and 30.

The turn motif peptide L803F 28 (KEAPPSPPQS(p)PF) (Fig. 14) inhibits GSK-3β, an isozyme of the serine-threonine kinase GSK-3 targeted in diabetes, neuronal development and neurodegenerative diseases.^57,58^ The peptide was based on the interacting section of heat shock factor-1 (HSF-1) and GSK-3. GSK-3 and GSK-3β recognise a phosphorylated residue, usually serine, in the sequence of SXXXS(p), that is docked in a positively charged binding pocket.^57^ Modelling also highlighted key hydrophobic interactions of F12 and proline residues in 28 engaging F93 at the edge of the binding pocket of GSK-3β.^57^ Pharmacophore based virtual screening identified 137 hits using the highlighted properties of the L803F peptide. By using an *in vitro* GSK-3 kinase assay, six compounds were identified as GSK-3β inhibitors with IC_50_ values ranging from 1–20 μM in an *in vitro* GSK-3 kinase assay.

Molecular docking of these compounds showed overlapping with the original peptide including the anthracenone-isoxazole core forming π–π interactions with F93 in GSK-3β. Interestingly, the carboxylic acid of 29, and the phenol of 30 were predicted to engage the positively charged binding pocket normally occupied for the phosphorylated amide, with the phosphoric acid group interacting with a second polar binding site (Fig. 14). 30 was shown to inhibit cellular GSK-3, demonstrating the rational design of small molecules from protein binding models.^59^

**Fig. 15:**
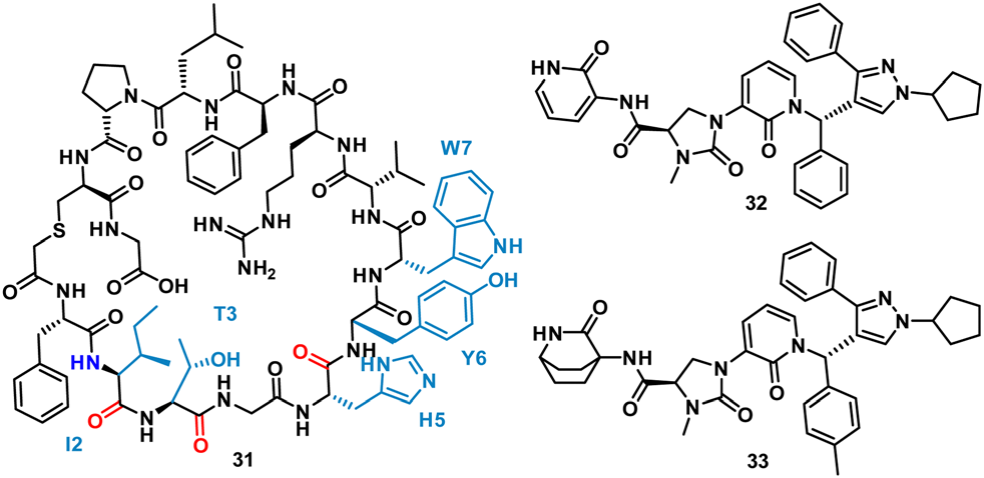
Structures of cyclic peptide 31 highlighting I2-W7 sequence (cyan), inspired hit 32, and subsequent small molecule 33.

Using mRNA display, Yoshida *et al.* identified the macrocyclic peptide 31 (FITGHYWVRFLPC*G – *cyclised sidechain to head *via* a thioether bond, Fig. 15) as a potent inhibitor of β-herpesvirus proteases in Human cytomegalovirus (HCMV, IC_50_ < 0.076 μM) and human herpesvirus 6 (HHV6 IC_50_ < 0.076 μM), but is unable to penetrate the cell. HCMV protease (HCMV^Pro^) has a catalytic triad consisting of Ser132, His63 and His157, and is activated upon formation of a homodimer.^60^ HHV6^Pro^ has a high structural similarity based on homology models.^61^

**Fig. 16:**
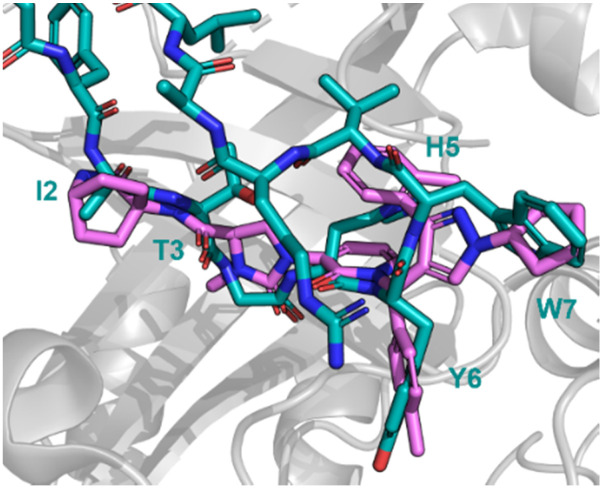
Peptide 31 (cyan, PDB: 8J3S) and 33 (magenta, PDB: 8J3T) bound to HCMV^Pro^. Key binding residues I2, T3, H5, Y6, and W7 are mimicked in 33.

Peptide 31 binds to these proteins in a wide and shallow active site which had proven exceptionally challenging for non-covalent small molecules, with the authors reporting no hits from a high throughput screen with 10^5^ compounds. The crystal structure of 31 with HCMV^Pro^ and an alanine scan highlighted Ile2, Thr3, His5, Tyr6 and Trp7 as key for binding (Fig. 16). These observations led to a virtual compound design looking for molecules able to mimic the hydrophobic sidechains of Ile2, Tyr6 and Trp7, hydrogen bond acceptors of the Ile2, Thr3 and His5 carbonyls, and hydrogen bond donors of the Ile2 NH. This culminated in compound 32 (Fig. 15), which satisfied the pharmacophore features, and upon synthesis demonstrated weak inhibitory activity for HCMV^Pro^ (32% inhibition at 99 μM) and HHV6Pro (IC_50_ = 18 μM). Encouraged by the proof-of-concept for compound design further structure based drug design, based on the virtual model of 31 led to the substitution of the pyridine ring for the 2-azabicyclo[2.2.2]octan-3-one, and the *p*-toluenic aromatic group, yielding compound 33 showing potent protease inhibition (HCMV^Pro^ IC_50_ = 2.5 μM, HHV6^Pro^ IC_50_ = 0.33 μM).

The new small molecule inhibitor, 33, is less than half the molecular weight of the starting peptide, 31, with drastically improved cell permeability, representing the first noncovalent small molecule binder at the active site of HCMVPro.^61^

### Mimicking peptide side chain interactions to enhance small molecule binders

**Fig. 17:**
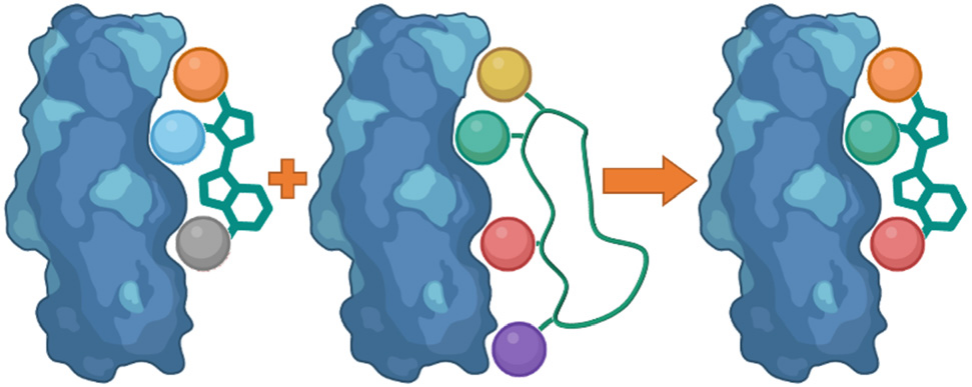
Using peptide side-chain interactions to improve modify known small molecule binders.

One of the main challenges faced by small molecules in targeting a PPI is controlling the large interface. Often interfaces have “hotspots” which can be identified when analysing how a peptide binds to the protein *via* crystal structures or modelling. The peptide SAR offers valuable information about what interactions can be modified and then applied directly to a small molecule inhibitor design (Fig. 17). This can inform new compound design or allow for improvements on known binders. This allows for SAR studies to be performed on the synthetically accessible peptides, and then applied to the synthetically challenging small molecules.

**Fig. 18:**
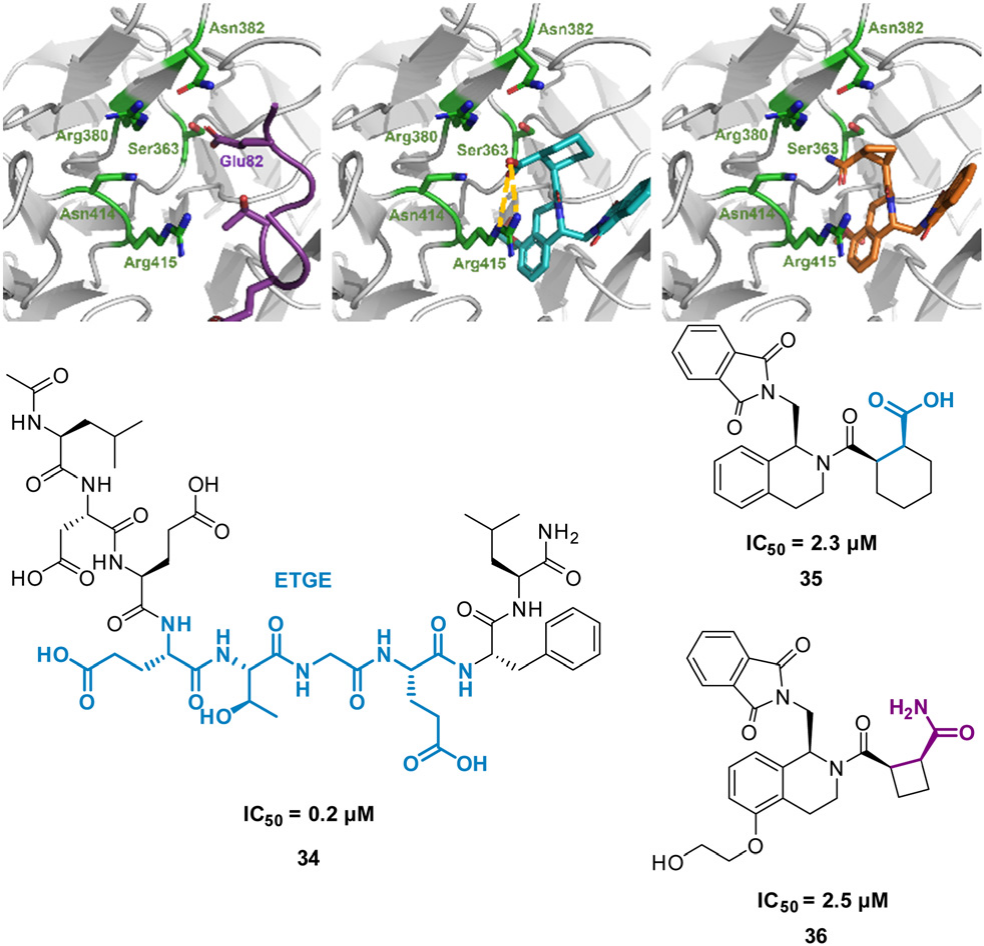
(left) 34 (purple, PDB: 2FLU) (centre) 35 (cyan, PDB: 35) (right) 36 (orange, PDB: 6SP4) interacting with KEAP1. Carboxylic acid interaction with Arg415 shown in yellow. Structures of turn-motif peptide 34, and small molecules 35 and 36.

Harper *et al.* demonstrated this approach when altering known small molecule inhibitor of the NRF2/KEAP1 interaction, tetrahydroisoquinoline 35 (IC_50_ 2.3 μM) into a non-acidic entity.^62^ Acid containing compounds have poor blood – brain barrier (BBB) penetration, undesirable for reaching the central nervous system, recent developments have shown replacing the acidic groups with primary amides can form a non-acid inhibitor of NRF2/KEAP1.^63,64^ The 9mer peptide LDEETGEFL 34 is a high affinity KEAP1 binder, derived from the NRF2 binding interface. The turn sequence _79_ETGE_82_ is key for binding, and Glu82 make key interactions into the P2 pocket of KEAP1 (Fig. 18(A)).^65^ This pocket is defined by the residue Ser363, Asn380, Arg382, Asn414, and Arg415. This site is also where the carboxylic acid of 35 is known to bind, interacting with Arg415 (Fig. 18(B)).^62^ To explore the necessity for an acidic functional group in this pocket peptide analogues were tested with Glu82 replaced with alkyl, aromatic and amide side chains. The SAR discovered that substitution with cyclobutylalanine produced an active peptide only 10-fold weaker than the original peptide despite lacking the acidic side chain functionality. When docking these peptides, superimposed images with 35 showed the cyclobutyl group of the peptide occupied the same region as the cyclohexyl region of the 35. Based on these observations, a series of 5-substituted tetrahydroisoquinolines containing carboxamide substituted cyclobutylamides resulted in 36 showing IC_50_ of 2.5 μM proving an alternative binding mode at the P2 pocket (Fig. 18(C)), engaging Asn414 and Ser36. This non-acidic 36 analogue can inhibit the PPI between NRF2 and KEAP1 in the low micromolar range.

**Fig. 19:**
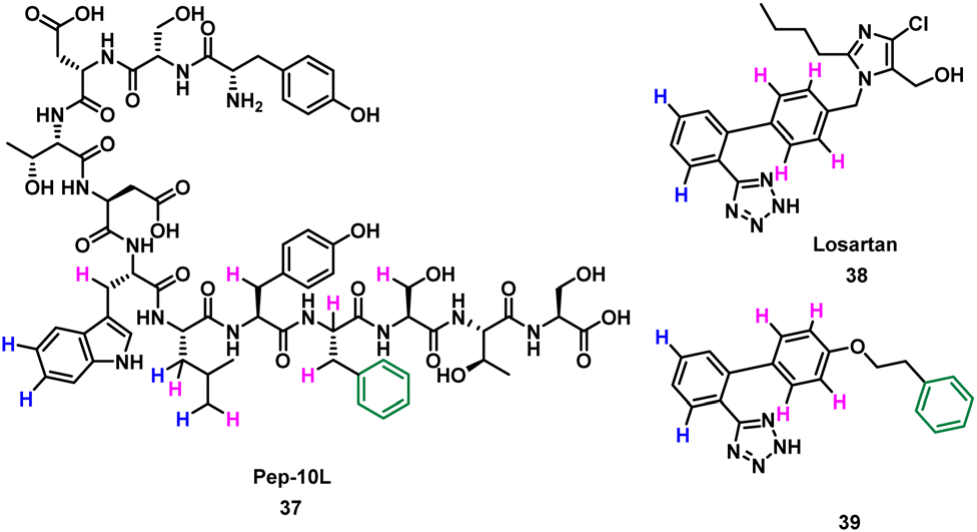
Structure of turn-motif-peptide 37, and small molecules 38 and 39. Hydrogen atoms highlighted blue occupy the same site, and those highlighted pink occupy the same site. The phenyl ring (green) was not accounted for in 39.

Takahashi *et al.* described a method of applying overlapping and non-overlapping sections of peptide and small molecules binders to improve small molecules, demonstrated with the platelet receptor glycoprotein VI (GPVI) and collagen.^66^ Using an NMR technique termed INPHARMA (protein-mediated interligand NOEs for pharmacophore Mapping),^67^ they identified the common pharmacophore of a peptide and small molecule that compete for the same binding site.^67–69^ Peptide pep10L 37 (Fig. 19) was identified through phage display to inhibit the GPVI–collagen interaction (*K*_D_ = 57 μM), binding to GPVI with a turn motif.^66^ The side chains of Trp6, Leu7, and Phe9 form a hydrophobic cluster and are key to GPVI binding.^66^ The peptide 37 binding site overlaps with the binding site of losartan, a small molecule inhibitor of aggregation of platelets, which relies on the phenyltetrazole for binding.^70^ Using the INPHARMA method the overlapping sections of peptide 37 and losartan 38 were identified, showing the phenyl group in losartan corresponded to the centre of the hydrophobic cluster in 37. The hydrogen atoms on the phenyltetrazole moiety occupied the same site as the hydrogen atoms on Trp6 and Leu7 (Fig. 19, blue). The hydrogen atoms on the central phenyl ring overlapped with hydrogen atoms on Trp6, Leu7, Tyr8, Phe9 and Ser10 (Fig. 19, pink).^67^ Notably, there was no overlap for the aromatic ring of Phe9 of 37 and 38. Addition of a phenyl ring to 38 to mimic Phe9 resulted in 39 (*K*_D_ = 52 ± 4 μM).^66^ The INPHARMA experiments confirmed that the new phenyl ring of 39 mimics Phe9 while retaining previous overlapping of the phenyltetrazole moiety and Trp6 and Leu7. This novel ligand based strategy has proven a successful way to modify pharmacophores using peptide structural binding information.^71^

## Conclusion

In conclusion, the development of small molecules that can mimic the inhibitory effects of cyclic peptides and turn motif peptides on protein–protein interactions holds great promise. The literature describes three common approaches for the transfer of the characteristics of cyclic or turn peptides to small molecules. The most used technique is the decoration of small molecule scaffolds with peptide functionality. This offers several advantages. Conceptually, this is the most straightforward, particularly when structural information is available. The small molecule scaffold can be selected to balance any physicochemical properties of the side chain functionality. The use of computational modelling and SAR has enabled the creation of small molecules with similar or enhanced pharmacological properties. The hits that are generated with this method can then be optimised with classical small molecule medicinal chemistry approaches. This method has yielded several examples of compounds that surpass the peptide that inspired them.

Recent advances in techniques offers promise for an increase in the design of small molecules from cyclic peptide starting points. Cyclic peptide development techniques, including phage display and mRNA display allow for rapid identification of peptides able to control a target protein. The development of analytical NMR techniques, including STD NMR and INPHARMA, allow the collection of structural information for more protein targets in a timely manner. These analytical techniques identify pharmacophores from multiple binding molecules and identify the key binding motifs to be included in small molecules.

The field of small molecule peptide mimetics is rapidly evolving, and we can expect to see further advances in the design and synthesis of small molecules that can mimic the biological activities of cyclic peptides and turn motif peptides.

## Conflicts of interest

There are no conflicts to declare.

## Supplementary Material
